# 

**Published:** 1896-11-14

**Authors:** 


### EARLY EDITION.

**Notices of Births, Deaths, and Marriages** are inserted in this column at a charge of 2s. 6d. for an announcement not exceeding four lines.

**Death.**

On November 4th, at Woodside, King Edward, Aberdeenshire, of rheumatism, NURSE COX, third daughter of the late Alexander Cox, farmer, aged 25. The deceased served her probationership in the Royal Infirmary, Perth. (1,085)

### NOTICE TO CORRESPONDENTS.

All MS., Letters, Books for Review, and other matters intended for the Editor should be addressed THE EDITOR, "THE HOSPITAL," THE HOSPITAL BUILDING, 28 & 29, SOUTHAMPTON STREET, STRAND, W.C.

The Editor cannot undertake to return rejected MS., even when accompanied by stamped directed envelope.

**Notice to Advertisers and Subscribers.**

All Advertisements, Orders for Copies of THE HOSPITAL, and Business Communications should be addressed to **THE MANAGER, (Not to The Eitdor),** THE HOSPITAL, The Hospital Building, 28 & 29, Southampton Street, Strand, London, W.C.

The Cost of Subscription, post free, is as follows:—Per week, 2½d.; per quarter, 2s. 8d.; per half-year, 5s. 3d.; per annum, 10s. 6d. Foreign:—Per annum, 15s. 2d.: payable, in all cases, in advance.

**IMPORTANT NOTICE.**

The EDITORIAL and PUBLISHING OFFICES of "THE HOSPITAL" are now TRANSFERRED to their new premises, THE HOSPITAL BUILDING, 28 & 29, SOUTHAMPTON STREET, STRAND, LONDON, W.C.

**NOTICE.—Vol. XX. of "The Hospital" (April to September, 1896), in handsome cloth binding, is now on sale at the Office of this Journal, price 7s. 6d. Cases for binding the Numbers contained in this Volume 1s. 6d. Index to Vol. XX., 2½d., post free.**

**The Monthly Part for November is now ready, Price 6d., by post 8d.**

### Scraps and Gleanings.

There is still a balance of £4,000 to be raised before the Home for Destitute Crippled Children at Wallsend can be cleared from debt.

The largest amount realised on Hospital Saturday in Bradford has been £2,319, but it is hoped that this year's collection may amount to £3,000.

Twenty years ago the amount expended on beer, wine, and spirits at the Leeds Hospital for Women and Children was £79 odd; last year it was £1 12s.

The rate of mortality among the patients at the Queen Charlotte's Lying-in Hospital during 1895 was in-patients 5·3, and out-patients ·73 per 1,000.

The appointment of a collector at a fixed salary in place of commission has resulted, at the Ayr County Hospital, in an increase in annual subscriptions.

The annual collections made by the Friendly Societies at Chelmsford on behalf of the infirmary show an increase of about £13 over the previous year.

Upwards of £33,000 has been promised towards the new infirmary scheme at Newcastle. Between £100,000 and £200,000 will be required to complete the full scheme.

The Hospital Saturday collections at Colchester this year in aid of the Essex and Colchester Hospital, resulted in a total from establishments and street collections of £329.

It is hoped that the Rustington Convalescent Home, which is the splendid gift of Mr. Henry Harben, will be opened for the reception of patients in the course of a few weeks.

The Floating Hospital of St. John's Guild carried during the season of 1896, 46,253 women and children; over 700 sick children were treated without a single death taking place on board.

The street collection in Cork in connection with Hospital Saturday amounted this year to £232 odd. Many halfpennies and farthings were received in the collection, as well as £18 in gold.

The proposed nurses' home at the Worcester Infirmary, the plans for which have been approved by the House Committee, is to contain 32 beds, while in cases of emergency many extra beds can be provided.

Five Hundred and Two in and 462 out patients were treated at the Fermanagh County Infirmary in the year 1895-6, the average cost of the former being returned at £2 0s. 7¾d., against £2 2s. 3d. in 1894-5.

Nearly all the furniture and fittings for the new buildings of the Glasgow Samaritan Hospital have been supplied by the ladies interested in the institution, who have made nearly every article of the napery.

The proposal of the Warrington Corporation to erect a hospital for the reception of small-pox patients at Houghton Green is being, despite the need of such a hospital, much opposed by the inhabitants of the district.

The first collection of the New York Hospital Saturday and Sunday Fund in 1879 amounted to $26,455 (say £5,291). Last year (1895), $58,919 (say £11,784) was collected, a sum which has only been beaten twice, viz., $60,264 in 1891, and $63,230 in 1892.

The sum of £14,500 is annually needed to keep in a state of efficiency the four charities of Bradford in which the Hospital Saturday Fund of that town is interested, viz., the Bradford Infirmary, the Eye and Ear Hospital, the Children's Hospital, and St. Catherine's Home.

Mr. Ben Evans has offered to defray the entire expense (about £750) of a new operating theatre for the Swansea Hospital, providing the mortuary is removed to a more suitable place. Meanwhile the present theatre at the Swansea Hospital is to be temporarily renovated.

The effect of an alteration in the wording of a notice exhibited in the waiting rooms of the Sheffield Public Hospital for Diseases of the Ear, Throat, and Skin, emphasising the fact that contributions by patients are purely voluntary, has been a reduction in the receipts from this source by about 10 per cent.

The receipts for the year 1895 at the Royal Surrey County Hospital, Guildford, when compared with those for 1894, show a slight falling off, the amounts being £4,944 and £5,082 respectively. Savings in expenses, however, have been effected, so that the expenditure has again been kept below the income.

Physicians in Russia consider it beneath their dignity to send an account to their patients, but leave it to them to pay what they think proper. This method can hardly prove remunerative to the physicians in some cases, nor can it, we should think, be much appreciated by the majority of patients.

### THE STUDENT OF MEDICINE.

### APPOINTMENTS.

W. E. Alderson, M.B., B.S.Durh , appointed Assistant Medical Officer to the Newcastle-on-Tyne Dispensary. R. H. Ashwin, L.R.C.P., M.R.C.S., appointed Assistant House Surgeon to the County Hospital, York. H. T. S. Aveline, M.R C S., L.R.C.P.Lond., Senior Assistant Medical Officer of the Bristol City Lunatic Asylum, Fishponds, appointed Medical Superintendent of the new Somerset County Asylum, Cotford, near Taunton. R. D. Boase, L.R.C.P.Lond., M.R C.S., appointed Medical Officer of Health to the Penzance Port Sanitary Authority. W. H. Brodie, M.D.Edin., D.P.H., appointed Medical, Officer for the First and Second Districts of the Battle Union. J. L. Burch, M.D., M.R.C.P., appointed Assistant Physician to the North London Hospital for Consumption, Mount Vernon. W. Chapman, M.R.C.S., L.R.C.P., appointed House Surgeon and Secretary to the Tunbridge Wells General Hospital. G. W. Chaster, M.R.C.S.Eng., L.R.C.P.Lond., appointed Honorary Assistant Medical Officer to the Southport Infirmary. H. S. Cooper, M.R.C.S., L.R.C.P., appointed Medical Officer of Health to the Brightlingsea Urban District Council. M. Farrant, M.R.C.S., L.R.C.P.Lond., appointed Medical Officer of Health to the St. Thomas Rural District Council. A. Greenwood, M.B , Ch B Vict., L.R.C.P.Edin., L.R.C.S.Edin., appointed House Surgeon to the Hulme Dispensary, Dale Street, off Stretford Road, Manchester. Mr. K. D. B. Hobbs, appointed Medical Officer for the Tutbury District of the Burton-upon-Trent Union. B. T. Lowne, M.R.C.S.Eng., L.S.A., appointed Medical Officer for the Third District of the Hartley Wintney Union. A. Macgregor, M.D , appointed Assistant Physician to the North London Hospital for Consumption, Mount Vernon. E. G. A. Morshead, M.D.Brux., L.R.C.P. Lond., M.R.C.S., appointed District Medical Officer to the Guildford Union. F. W. H. Robson, M.B., C.M., appointed Assistant House Surgeon to the Rotherham Hospital. R. Trimble, L.R.C P., L.R.C.S.I.Edin., appointed Medical Officer for the No. 1 District of the West Bromwich Union. H. M. Wilson, M.D., appointed Chief Inspector of Rivers under the West Riding Rivers Board.

### PASS LIST.

### Triple Qualification Examination in Edinburgh.

The quarterly examinations of the Royal College of Physicians of Edinburgh, Royal College of Surgeons of Edinburgh, and Faculty of Physicians and Surgeons of Glasgow, for the triple qualification in Edinburgh took place in October with the following results:—

First Examination (4 years' course).—Of 10 candidates entered, the following 6 passed: H. V. Mayne, Cork; W. Ritchie, Perth; R. J. Morgan, County Antrim; W. Dunseith Dickson, Cookstown; D. P. O'Sullivan, Cork; and E. Hepworth Alton, Plymouth.

First Examination (5 years' course).—Of 33 candidates entered, the following 15 passed the examination: Fanny Wood, Kerry; J. E. Ratcliffe, Accrington; Prudence Elizabeth Gaffikin, Belfast; A. Graham, Peebles; M. H. Lynch, Galway; J. Noonan Meade, County Cork; G. Robertson, Dunfermline; W. Donovan, County Cork; H. E. C. K. Murray, Nova Scotia; F. W. K. Tough, Aberdeen; E. E. Evans, Bengal; D. P. Johnstone, Dunmanway; W. C. M. Burnside, Belfast; J. T. Moore, Dublin; and G. Kee, Greenock. Four candidates passed in one or two divisions.

Second Examination (4 years' course).—Of 49 candidates the following 21 passed the examination: Louise Blanche Smith, Calcutta; S. J. B. Fox, Sheffield; P. H. Hadfield, Birkenhead; N. Culliman, Cork; W. E. Heal, Halifax; W. B. K. Richards, Buckingham; P. H. du Plessis, South Africa; E. B. Anderton, Lancashire; J. MacRae, Beauley; D. Wilson, County Antrim; J. J. Scanlan, County Cork: J. S. Crabbe, Aberdeen; A. E. Crabbe, Aberdeen; R. H. Hunter, Repton; R. V. Donnellan, Limerick: H. N. Barnett, County Down; A. Galloway, South Shields; B. R. Roberts, Athlone; C. A. Mateer, County Down; R. D. C. Rose, Wigtown; and R. F. Huston, Belfast. Five candidates passed in one or two divisions.

Second Examination (5 years' course).—Of 29 candidates entered, the following 14 passed the examination: A. M. Hardie, Edinburgh; M. King, Ireland; E. Hill, Cork; D. M. Macgregor, Oban; G. D. Bateman, Cork; E. L. M. Hackett, Cork; D. Campbell, Argyll; W. Denness, Bothwell; J. T. Moore, Dublin; P. J. van Coller, Cape Colony; Jessie Jane Graham, Dunfermline; Lizzie Beatty, Ballymena; J. Buckley, Ashton-under-Tyne; and Emma Bond, Accrington.

### ROYAL NATIONAL PENSION FUND FOR NURSES.

*President*—**H.R.H. THE PRINCESS OF WALES.**

*Chairman*—WALTER H. BURNS, Esq. *Deputy-Chairman*—HENRY C. BURDETT, Esq.

**PENSIONS. SICKNESS. ACCIDENT.**

### Total Invested Funds, £300,000.

Nurses are invited to join the Fund on account of the substantial and exceptional advantages which it offers them and which *they cannot obtain elsewhere.* The following are the chief points:—

1. **The Fund is Mutual and essentially Co-operative.**

2. **Easy Payment of Premiums.**

   Nurses can pay their premiums monthly or otherwise as best suits their convenience.

3. **The Fund is open to every Nurse.**

   Nurses can assure for Pensions of any amount commencing at any age.

4. **An Investment and Savings Bank.**

   Those entering under the returnable scale can have their premiums returned to them with compound interest, less a small deduction for working expenses. Interest allowed on deposits.

5. **Additions to Pensions.**

   Besides the Pension entered for, substantial additions thereto may be anticipated in the shape of bonuses.

6. **Sickness and Accident Assurance.**

   Policies are issued in connection with Pension policies assuring 10s., 15s., or 20s. a week in cases of incapacity from work through sickness or accident.

**Full particulars to be obtained from THE SECRETARY,**
ROYAL NATIONAL PENSION FUND FOR NURSES,
28, FINSBURY PAVEMENT, LONDON, E.C.

### CHARTS

**PRICES (Post or Carriage Paid)**—
1,000, 25s.; 500, 13s. 6d.; 250, 7s. 6d.; 100, 3s. 6d.; 50, 2s.; 25, 1s. 3d.

### TEMPERATURE NURSING AND DIET CHARTS,

As Used in almost all our principal Hospitals.

**THE BEST AND CHEAPEST PUBLISHED.**

WODDERSPOON & CO., 7, SERLE STREET, LINCOLN'S INN, W.C.

